# Insights from Characterizing Extinct Human Gut Microbiomes

**DOI:** 10.1371/journal.pone.0051146

**Published:** 2012-12-12

**Authors:** Raul Y. Tito, Dan Knights, Jessica Metcalf, Alexandra J. Obregon-Tito, Lauren Cleeland, Fares Najar, Bruce Roe, Karl Reinhard, Kristin Sobolik, Samuel Belknap, Morris Foster, Paul Spicer, Rob Knight, Cecil M. Lewis

**Affiliations:** 1 Department of Anthropology, University of Oklahoma, Norman, Oklahoma, United States of America; 2 Department of Computer Science, University of Colorado, Boulder, Colorado, United States of America; 3 Department of Chemistry and Biochemistry, University of Colorado, Boulder, Colorado, United States of America; 4 Department of Chemistry and Biochemistry, Advanced Center for Genome Technology, University of Oklahoma, Norman, Oklahoma, United States of America; 5 School of Natural Resources, University of Nebraska, Lincoln, Nebraska, United States of America; 6 Climate Change Institute and Department of Anthropology, University of Maine, Orono, Maine, United States of America; University of York, United Kingdom

## Abstract

In an effort to better understand the ancestral state of the human distal gut microbiome, we examine feces retrieved from archaeological contexts (coprolites). To accomplish this, we pyrosequenced the 16S rDNA V3 region from duplicate coprolite samples recovered from three archaeological sites, each representing a different depositional environment: Hinds Cave (∼8000 years B.P.) in the southern United States, Caserones (1600 years B.P.) in northern Chile, and Rio Zape in northern Mexico (1400 years B.P.). Clustering algorithms grouped samples from the same site. Phyletic representation was more similar within sites than between them. A Bayesian approach to source-tracking was used to compare the coprolite data to published data from known sources that include, soil, compost, human gut from rural African children, human gut, oral and skin from US cosmopolitan adults and non-human primate gut. The data from the Hinds Cave samples largely represented unknown sources. The Caserones samples, retrieved directly from natural mummies, matched compost in high proportion. A substantial and robust proportion of Rio Zape data was predicted to match the gut microbiome found in traditional rural communities, with more minor matches to other sources. One of the Rio Zape samples had taxonomic representation consistent with a child. To provide an idealized scenario for sample preservation, we also applied source tracking to previously published data for Ötzi the Iceman and a soldier frozen for 93 years on a glacier. Overall these studies reveal that human microbiome data has been preserved in some coprolites, and these preserved human microbiomes match more closely to those from the rural communities than to those from cosmopolitan communities. These results suggest that the modern cosmopolitan lifestyle resulted in a dramatic change to the human gut microbiome.

## Introduction

The human distal gut is a complex bacterial bioreactor housing a 100 times the number of genes than its human host genome [1] and functions as a vital adaptive “organ” [2]. The genomics of microbial ecologies (microbiomes) has gained great attention recently, in part, because the Human Microbiome Project (HMP) a U.S. National Institutes of Health Initiative [3]. One primary objective of the HMP is to determine whether there are core aspects of microbiomes shared by healthy humans. One consideration is that core aspects of microbiomes observed in modern cosmopolitan populations today may underrepresent core aspects of human microbiomes that had existed historically, or prehistorically.

The modern cosmopolitan transformation, such as the advent of processed foods, antibiotics and other systemic drugs, and various sanitation technologies, has impacted our interaction with microbes. This transformation has reduced the spread of aggressive infectious diseases, which are chiefly problematic for the densely populated populations. Unfortunately, these interventions are far from targeted strikes, and a wide range of potentially beneficial microbes are caught in the crossfire [4]. Analogous to James Neel's hypothesis regarding syndromes of impaired genetic homeostasis [5], our modern lifestyle may have impacted ancestral mutualistic relationships between humans and microbes. The result is a potential increased risk for autoimmune diseases among other health related conditions [4], [6], [7], [8], [9], [10].

Understanding the evolution of human-microbe ecosystems greatly benefits from a baseline reflecting an ancestral state of the human microbiome. The study of our closest living cousins, the other great apes, provides one path to reconstruct ancestral microbiomes. But the human-chimp common ancestor was over 6.5 million years ago, providing ample time for extensive evolution in the human line. Alternatively, the study of modern people living a more traditional and isolated lifestyle provides a valuable perspective on the ancestral state of human microbiomes, but arguably, there are no traditional communities unaffected by modern globalization and even if we make exceptions for those communities deep within South American jungles, these communities provide a very restricted view of the potential variation in ancestral microbiomes recovered from other environments.

Retrieving human microbiome information from samples left behind by our distant ancestors would provide an ideal approach to understanding the coevolution of humans and microbes. Fecal material is the typical sample proxy for characterizing distal gut microbiomes. Therefore, ancient fecal samples (coprolites) have the potential to reveal the ancestral state of the human gut microbiome [11], are common within some archaeological sites representing sedentary lifestyles, as well as for some hunter-gatherer sites where coprolites have been retrieved from cave deposits and from mummies. Ideally, coprolites provide a view of how humans and microbes coevolved in response to different environments over time, including responses to both natural and cultural change. Previous molecular analyses of coprolites have been used to retrieve dietary information [12], [13]. However, the potential for retrieving ancient microbiome data is confounded by continuing microbial activity, environmental contamination, degradation and other post-depositional processes.

We provide a systematic examination of coprolite microbial communities from three different archaeological sites, each exposed to different environmental conditions. We assess the challenges of ancient gut microbiome research attributed to post-depositional processes including molecular degradation and contamination, and we retrieve ancient microbiome profiles consistent with the primate gut. The results suggest that there are aspects of ancestral human microbiomes that are atypical of modern cosmopolitan populations, and they reveal novel avenues to explore the prehistoric human condition.

## Results

**Figure 1:**
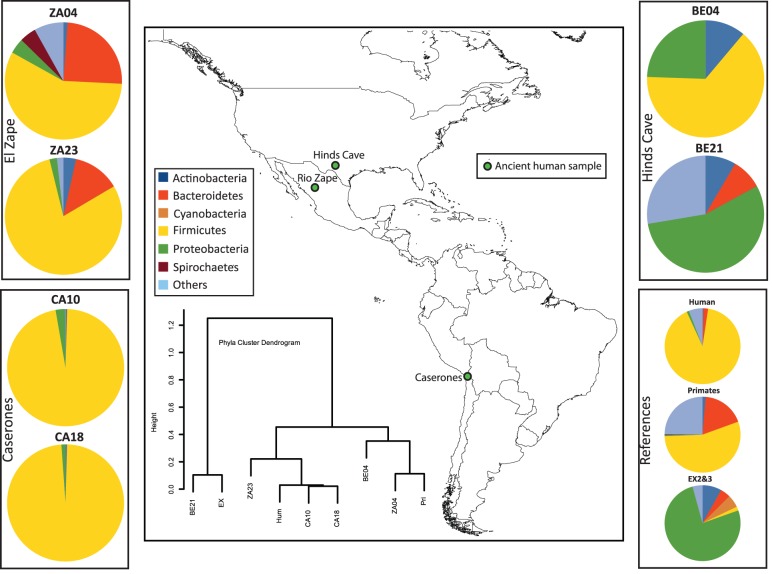
The geographic distribution and bacterial diversity of the included samples. These data resulted from comparison of the 16S rRNA V3. Taxon distribution and cluster dendrogram were limited to phyla with a frequency of 5% or more.

We analyzed the microbial composition of two coprolite samples from each of the three archaeological sites: Hinds Cave (∼8000 years B.P.) in southwestern United States, Caserones (1600 years B.P.) in northern Chile, and Rio Zape in northern Mexico (1400 years B.P.) shown in Figure 1 (see Figure S1 for an overview of methods). These three sites provide a broad range of environmental conditions. Hinds Cave is a rock shelter with extensive and repeated human occupation for thousands of years. The Hinds Cave coprolites (BE04 and BE21) were morphologically intact and part of abundant geological lenses of coprolites found throughout the site [14]. In contrast, the Caserones coprolites (CA10 and CA18) were retrieved directly from the intestines of a mummy and had no exposure to soil. The coprolites from Rio Zape (ZA04 and ZA23) were recovered from the La Cueva de los Chiquitos Muertos [15], a deep, dry cave. The Rio Zape coprolites were originally deposited in a midden composed of sand and refuse in the cave. The midden was used for seven child burials, which were made at, or around, the same time. The midden with the burials, refuse, and coprolites were sealed under an adobe layer that prevented disturbance. The intact preservation of material under the adobe layer included food offerings of agave, beans, corn, cucurbits and piñón.

Results for negative controls are included. EX02 refers to a negative control used during DNA extraction in which the coprolite sample was replaced by water. EX03 refers to a negative control used during amplification in which the DNA extract was replaced by water. Results for these controls required a greatly extended qPCR reaction than that used for the ancient DNA reactions (see Methods).

Figure 1 provides the geographic and phyla distribution for the ancient samples, in comparison to the phyla inferred from modern primates, while table S1 provides QIIME 1.3.0 [16] taxonomic assignments in detail. The coprolites from Rio Zape have phyletic representation that is consistent with that observed in humans and primates, while the coprolites from Caserones have very low diversity with respect to phyla. A higher phyletic diversity for Rio Zape samples compared to Caserones samples is observed in a species-level rarefaction analysis (Figure S2). Analysis of the Hinds Cave BE04 sample showed phyla typical of the gut. Sample BE21 harbored phyla observed in pooled negative controls, raising an initial concern about contamination, which was later resolved by additional analyses.

**Figure 2:**
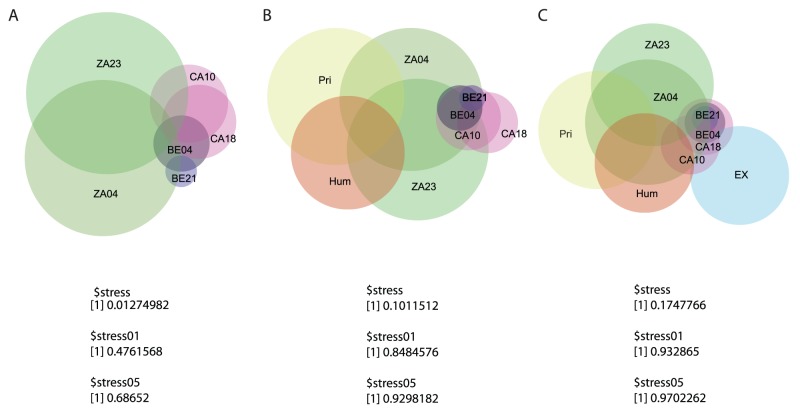
Venn-Euler diagram of OTUs at 97% pairwise identity representing 1,045 OTUs. The sizes of the circles and intersections are proportional to the number of OTUs listed and shared by each sample. Stress value for A is 0.01274982, and it increased in B and C as more samples are added. All the stress values are lower than the predicted value at 0.01 and 0.05, suggesting that the grouping is non-random.

Venn-Euler diagrams (Figure 2) provide a general pattern where coprolites from the same site tend to cluster. Specifically, the microbes present in the Rio Zape samples clustered together and represent constituents in the primate gut. In contrast, although the Caserones and Hinds Cave data were clustered among Rio Zape, they did not contained microbial similarities with primate gut as observed in Rio Zape. These Venn-Euler diagrams therefore reveal that the coprolites from the same site shared a more similar coprolite microbiome than those from different sites and that the coprolites varied greatly in their degree of similarity to the modern and primate gut. A Principal Coordinates Analysis (Figure S3) is consistent with Venn-Euler analysis; both approaches depict resemblances between Rio Zape and primate gut microbiome.

**Figure 3:**
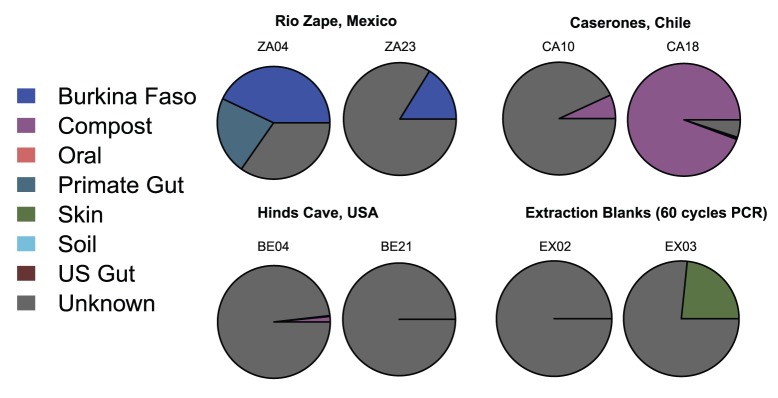
Bayesian source-tracking results for ancient coprolite samples. Both Rio Zape samples assign partially to the rural African children source, with sample ZA04 also containing a predicted partial match to the modern non-human primate gut source. Caserones sample CA18 assigns almost entirely to the compost source with relatively high confidence; sample CA10 is predicted with low confidence to contain a small proportion of compost (See Figure S5 for variability in proportion estimates). The sources for the Hinds Cave samples were unrecognized given our training data, resulting in nearly complete assignments to the “Unknown” source. When extraction blanks were subjected to 60 cycles of 16S PCR, the amplified microbial community signature assigns to either a skin community or unknown community.

**Figure 4:**
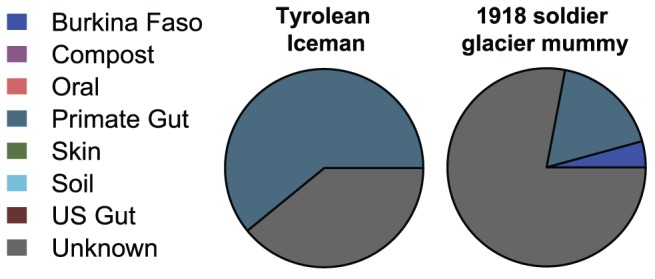
Bayesian source-tracking results for the Tyrolean Iceman and a 1918 soldier glacier mummy (sequence data published in [**19**]). The Tyrolean Iceman sample exhibits a substantial degree of similarity to a primate gut, while the soldier mummy assigns mostly to an unknown microbial community, within minor and low-confidence proportions assigning to the primate gut and rural African child gut sources (See Figure S6 for variability in proportion estimates).

To further assess how well the coprolites reflected a gut microbiome compared to other ecologies, we used SourceTracker [17], a Bayesian approach to estimating the proportion of well-characterized environments or “source communities” in a coprolite or “sink” sample. All studied coprolites included a high proportion of unknown sources, which is expected considering that there are few well characterized source communities publicly available for comparison. Figure 3 shows the source tracking results for the ancient samples and controls. Similar to Venn-Euler diagrams, source tracking analysis showed substantial variation among collection sites and generally more consistency between samples within sites. Most striking, both Rio Zape coprolites exhibited a gut microbiome signature with similarities to the children from a rural African village with the exclusion of a sample of U.S. modern adult gut microbiomes (see Figure S4 for a heat map of these data and Figure S5 for the variability in the source proportion estimates). ZA04 also harbored similarities to non-human primate gut. The coprolites from Caserones and Hinds Cave showed little similarity to a gut microbiome environment. A portion of Caserones coprolite microbial community was similar to compost, which may be explained by the post-mortem gut serving as an organic bioreactor filled with carbon and nitrogen from decaying food detritus. The microbial community assignment for Hinds Cave failed to assign well to any source environment. These results were obtained by merging the various source and sink data using species-level taxonomy assignments; a similar analysis using operational taxonomic units (OTUs) picked by reference against the February 4^th^ 2012 Greengenes [18] reference database at a level of 97% sequence similarity produced negative results, presumably due to the use of different 16S rRNA regions and protocols in the various source and sink data sets. The negative controls assigned to human (U.S. modern) skin and unknown environments. Importantly, none of the ancient samples included skin as a significant source, which provides additional confidence that laboratory contamination within ancient samples was limited. Similar to the Rio Zape samples, SourceTracker analysis [17] on the intestinal coprolite data for the Tyrolean Iceman (Ötzi the Iceman) and an Austrian soldier killed in 1918 and retrieved from a glacier [19] had assignments to the rural African children and to non-human primates and excluded assignments to the U.S. adult modern gut microbiome (Figure 4), although there was high variability in the source proportion estimates of the Austrian soldier (Figure S6).

Bacterial taxa of special interest to understanding the human host were further screened. *Bifidobacterium* was present in ZA23 (Figure S7), which include species typical of breast feeding infants and children. ZA23 also harbored abundant Prevotella (Figure S8), consistent with the proposedhuman enterotype [20]. The ZA04 sample harbored spirochaetes matching *Treponema berlinense* (NR_042797.1) with a Blastn E value of 4e-75, query coverage of 100% and Max identity of 99% (158 bases of 159). Similar spirochaetes were observed in children from a rural African community, but are atypical of children and adults from cosmopolitan populations [21].

During the time this publication was in peer review, additional data for two extant rural populations were published: the African Malawi and a South America population from Venezuela [22]. We conducted a source tracking analysis for the Rio Zape data using adults from these new data and data for primates and soils (Figure S9). The results were nearly identical to those presented in Figure 3; however, instead of the rural sample from Burkina Faso providing the major source, it was either the rural sample from Malawi or Venezuela providing the major assignment (Figure S9). The rural sample from Malawi or Venezuela also provide the major assignment when we partitioned the human data into age groups (Figure S10); although in these latter analyses, there were minor assignments to US adult for both Rio Zape samples and a minor assignment to US infant for sample ZA23.

## Discussion

Recovery of information about the ancestral state of the distal gut microbiome from coprolite samples is feasible, which is well demonstrated by the fact that the results from Rio Zape cave deposit were consistent with the pattern observed in rare and pristine samples retrieved from permafrost mummies. Soil contamination, an obvious concern for most coprolite studies, is a manageable barrier when applying appropriate tools such as SourceTracker [17]. The ability to retrieve gut microbiome data from coprolite samples provides an exciting new line of evidence for reconstructing a past lifeway.

Not all coprolites are expected to retain human microbiome information. It is unclear whether the results from Hind's Cave and Caserones reflect different preservation conditions, depositional process, or unique gut microbiomes. For future research, a characterization of the microbiome of these samples using smaller amplicons or a greater depth of sequencing will provide important insights.In the meantime, it clear that some coprolites, like those from Rio Zape, provide well preserved human gut profiles.

Our data from Rio Zape provides examples of biographical information. In figure S10, ZA23 had a partial assignment to US infant. *Bifidobacterium breve* is the most abundant *Bifidobacterium* in ZA23. *Bifidobacterium* has been found to be more prevalent in children, especially in younger children who are breastfed, and *B. breve* is almost exclusively found in breastfed infants [21]. *B. breve* is nearly absent, observed only once in one sample, in a study of non-human primates [23]. These results lend support for the claim that ZA23 is from a child. Moreover, our sample from Rio Zape harbors abundant *Prevotella* which is associated with a diet rich in carbohydrates [24]. These observations at Rio Zape are similar to a pattern observed within children from rural Africa who have both *Bifidobacterium* and high *Prevotella* [21]. The coprolite from Rio Zape is more consistent with that of a child than an adult; corroborating this inference is the fact that the burials at Rio Zape are seven children [15]. Although this association is not definitive, the potential use of bacterial genera to characterize specific human lifestyles or life states has exciting ramifications. As human microbiome research continues to mature, we expect it will contribute greatly to the fields of archaeology and forensics.

Information from Rio Zape also supports a current hypothesis about the composition of human microbiomes in traditional communities, potentially revealing an important aspect of the ancestral human microbiome. Spirochaetes are atypical of gut microbiomes in cosmopolitan communities. However, *Treponema* was reported by Filippo et al. [21] in their comparative study of modern microbiota in children from Europe and rural Africa. In their study, *Treponema* was observed in the rural African children but was absent in the European children. They hypothesized that the *Treponema* may enhance the hosts ability to extract nutrients from fibrous foods and may provide anti-inflammatory capability. They raise the hypothesis that microbiota coevolved with ancient diets and that changes in food production greatly impacted the intestinal microbiota. *Treponema* was also observed in the published rural data for Malawi and Venezuela [22]. The results from Rio Zape provide further support for *Treponema* as part of the rural human microbiome. Specifically, *Treponema* now is observed in four rural communities from different continents, three extant communities and one community that has been extinct for over a thousand years.

In conclusion, ancient coprolite microbiomes can be retrieved to analyze the bacterial phylotypes. The analyses suggest that ancient microbiomes are different than the current cosmopolitan human microbiomes and are more similar to rural microbiomes. Our results suggest that the most dramatic change to the gut microbiome in the human ancestral line has been the modern transformation of the human condition in cosmopolitan populations.

## Materials and Methods

### Samples

The novel data originated from six paleofecal samples from three different archeological sites:

1. “La Cueva de los Chiquitos Muertos”, an archaeological site near Rio Zape, Durango, Mexico (Rio Zape), dating to 1400 B.P. These samples were collected during excavations by Richard and Sheilagh Brooks in the 1960s and stored in sterile forensic specimen bags in a cool and dry place at the University of Nebraska State Museum. In 2007, these samples were sent to the Molecular Anthropology Ancient DNA Laboratory at the University of Oklahoma.
2. “Hind's Cave” Texas, dating to ∼8000 years B.P. These samples were collected from an Archaic habitation in the lower Pecos of region of Texas. They were stored in specimen bags and plastic containers in a cool and dry place at the University of Maine's Department of Anthropology. In 2008, these samples were sent to the Molecular Anthropology Ancient DNA Laboratory at University of Oklahoma.
3. Caserones, Chile, dating to 1600 B.P. The samples are from two females aged approximately three and five years old, respectively. Both females had perimortem cranial fractures, suggestive of the cause of death. These samples were retrieved directly from the naturally mummified gut tissue, having no direct exposure to soil. The samples were stored in specimen bags at the University of Minnesota, Duluth. In 2008, these samples were sent to the Molecular Anthropology Ancient DNA Laboratory at University of Oklahoma.

These samples were processed in a positive pressure clean-room, with isolated ventilation where incoming air passes through ISO 7 (class 10,000) HEPA-filtration system. The room is equipped with UVC lighting. Sterile disposable gowns, gloves, hair nets and masks were worn at all times while working on these samples.

### DNA extraction and purification

A layer of around 1 cm from each coprolite's surface was removed with a sterile scalpel to remove contamination from previous handling. Between 0.11 and 0.22 grams of each coprolite's interior matrix was used for each DNA extraction. DNA extraction was performed using the UltraClean Fecal DNA Isolation Kit (MOBIO), adding an extra wash step with the S4 solution. A negative control for the DNA extraction was included where sterile ddH20 was substituted for the DNA template. The control sample was processed with the coprolite samples following the same protocols. DNA was eluted from the filter in 50 µl of S5 solution. The sample and control were re-purified and concentrated with the MiniElute PCR Purification Kit (Qiagen) silica columns adding a second wash step with buffer PE and with a final elution volume of 20 µl of EB buffer. Purified DNA quantitation and quality control were performed following previously published methods [11].

### DNA authentication

The majority of the DNA fragments from the coprolites were below 200 bp in length. To further authenticate that our data represented ancient molecules, rather than modern contaminants, we compare the PCR success related to the size of the amplicon. Our results showed that V5-V6 region, from 784F and 1061R [25] (277 bp) produced much less PCR product than V3 region, from 341F and 529R [26](188 bp) which is consistent with results expected from degraded ancient DNA molecules rather than modern contaminants. Source tracking methods discussed later provided additional authentication. Cave coprolites were identified as human by their intact cultural context, microscopic components (such as maize), as well as characterizing the human, specifically Native American, mitochondrial DNA haplogroup. The Rio Zape coprolites were haplogroup B, and Hinds Cave BE04 was haplogroup C, all of which are Native American haplogroups. Hinds Cave BE21 sample has yet to provide haplogroup results but were in the same archaeological context as BE04.

### Amplification of 16S rRNA V3 region

Purified DNA was diluted at 1∶10 and 1∶100 using sterile ddH20. Quantitative PCR (qPCR) was used to assess potential inhibitors and quantify copy number of 16S rRNA V3 using published primers U341 and U529R [26]. Each qPCR reaction contained: 1X Platinum® PCR SuperMix High Fidelity PCR Buffer (Invitrogen), 170 nM of each primer (IDT), 0.1×SYBR (Molecular Probes) and 5 µl of DNA template for a total of 50 µl. In an 8-well PCR tube strip with individual caps, reactions included the DNA extract, the DNA extract after additional purification, the negative control for the DNA extraction, a negative control for the PCR reaction where sterile ddH20 was substituted for DNA template, and dilutions of the purified DNA extract to assess the extent of DNA preservation and to handle inhibitors. If qPCR results for dilutions did not follow the proportionality of a standard curve, the samples presented inhibitors. None of the samples showed this pattern. The temperature profile for the reactions included an initial activation of the enzyme at 94°C for 2 minutes, followed by 60 cycles of 94°C for 15 seconds, 54°C for 15 seconds and 72°C for 15 seconds. Melting curves were obtained measuring the fluorescent intensity of the PCR product in a linear denaturation ramp from 35 to 90°C, increasing 1.0°C every 6 seconds. All the qPCR reactions were set up in the ancient laboratory in order to avoid external contamination. Once the qPCR tubes were sealed in the ancient laboratory, they were brought to the modern DNA lab for amplification. The qPCR information was used to normalize samples to 10^4^ and 10^5^ copies per µl. The initial copies of 16S amplicons were the following 60 cycles were: BE04 = 1.13E+05, BE21 = 9.43E+05, CA10 = 2.94E+05, CA18 = 2.60E+05, ZA04 = 3.79E+05, ZA23 = 1.16E+06, EX03 = 3.60E+03, EX02 6.87E+02, Water control = 0.00E+00.

### Sample preparation for 454 pyrosequencing of 16S rRNA V3

Using normalized dilutions of template, the 16S rRNA V3 region from each sample was amplified by qPCR. Normalized samples were amplified in duplicate, tagged and then pooled before 454 library preparation. qPCR for sample preparation followed the protocol described above with the exception of using 30 cycles for the normalized ancient DNA solution and 60 cycles for the negative controls. Because of the few copies of 16S molecules in the negative controls (EX02, EX03), these sample could not be normalized. pPCR proceeded on the negative controls without normalization. The negative controls provided no evidence for amplification at 30 cycles. After 45 cycles, negative controls began to show amplification of contaminants. Because these contaminants reflect a greatly extended qPCR reaction, it is unlikely they have impacted our ancient DNA frequency data in any significant way, but they are included for full disclosure. The qPCRs for the negative controls were also performed in duplicate, tagged, and the pooled before library preparation. Different 454 adaptors carrying a 10-base barcode were added to the PCR products, and after pooling, were sequenced on a Roche 454 GS FLX Titanium pyrosequencer [15].

### 16S rRNA gene sequence analysis

Data was de-noised using the Pyrosequencing pipeline (http://pyro.cme.msu.edu/) to retrieve sequences by barcode, remove primers and provide quality filtering. Criteria for inclusion in our analyses required each sequence reads to have an exact barcode with exact primer sequences and a quality score over 25 [27]. After removing primer sequences, barcoded data from pyrosequencing of 16S rRNA amplicons averaged 150 bases in length. A minimum of 14,000 reads were generated per coprolite sample (Table S2). Published 16S rRNA V3 were aligned and trimmed to match our dataset: Gill *et al.* [1] and Ley *et al.* [28] as well as 399 other sequences from one human sample (NCBI accession numbers GU939195.1 to GU939593.1). The compiled dataset was analyzed with the software package Quantitative Insights Into Microbial Ecology (QIIME; http://qiime.sourceforge.net) using default settings with one modification: the length cutoff was set at 130 instead of the default 150 bases.

To infer taxonomic assignment and to provide rarefaction curve analysis, the screened data were analyzed using the software package Quantitative Insights into Microbial Ecology – QIIME 1.3.0 [16] using the default settings. The frequency of phyla were further characterized using hierarchical analysis using the *hclust* script in R [29].

These data were compared to those published from fecal samples from six different primates: one Bonobo (BNO), one Chimpanzee (Chimp), two Gorillas (GOR and GORSD), one Marmoset (ML) and one Orangutan (ORANG) [28] and two human individuals from Gill [1] and one retrieved from NCBI (accession numbers GU939195.1 to GU939593.1). The R function Venn-Euler [30] was applied to data with an OTU assignment at 0.97.

The ZA23 sample provided different OTUs belonging to the *Bifidobacterium* genus. These *Bifidobacterium* were compared to eight previously published datasets (NCBI accession numbers JN093131, AB186296, AY172657, HM856589, EF203955, HQ851039, JN180852 and AB507156) using a Neighbor-Joining method. The aligment and tree were generated using MEGA5 [31]. Data from Rio Zape and published data from children from rural Africa and Europe [21] were used for a comparative analysis of the frequency of the Prevotellaceae family. Data from all other taxa were pooled into the other category.

### Source tracking analysis

Bayesian microbial source tracking was performing using SourceTracker [17]. We combined data for source and sink data sets, which included sequence data for different regions of the 16S ribosomal gene, in two ways. First, we picked OTUs de novo at a level of 99% sequence similarity and binned the OTUs by species-level taxonomy assignment in QIIME [16]. After binning by taxonomy in each dataset separately, we combined the taxonomy tables for the various source/sink data sets. Second, we picked OTUs at a level of 97% sequence similarity to the February 4^th^ 2012 Greengenes [18] reference database. We modeled the coprolite samples as a mixture of the known environments using both the de novo-based species-level taxonomies and, separately, the reference-based 97% OTU table. For each sample, the estimated proportion of each source was drawn after 1,000 “burn-in” iterations using Gibbs sampling. We repeated the Gibbs sampling procedure for 25 random restarts, drawing one proportion estimate per restart. We used the empirical variation in mixture predictions from the 25 Gibbs sampling restarts to estimate confidence in the mixture estimates; the variation can be visualized directly (Figures S5 and S6). For each run, we rarified data for each coprolite at 10,000 sequences in the primary analysis (or fewer for samples with lower coverage). Rio Zape data had more sequences on average; however, rarifing these data further (500, 1000, 2000, 5000 sequences) does not change the interpretation of the results (example Figure S11). To avoid underestimating the Unknown environment source proportions (equivalently, to reduce the likelihood of false positive source assignments), we chose the SourceTracker hyperparameter α_2_ value of 0.00415 for the Unknown environment by re-estimating the source proportions after removing each one of the source environments from the training samples. We chose the largest α_2_ value that did not show evidence of identifiability concerns between the sources. In other words, we ensured that removal of one source (e.g. primate gut) from the training data did not cause the proportion of another source (e.g. modern Westernized gut) to increase. We used a similar approach to choose the α_2_ value of 0.01 for the iceman and Austrian soldier samples.

The source environment communities included microbiome from the U.S. human gut, oral and skin microbiomes of nine adults from Boulder, Colorado [32], human gut microbiome from 11 children of five to six years old from a rural community from Burkina Faso [19], 37 primate gut microbiomes [22], [33], one compost [34] and a representative set of 88 soils [35]. In addition, we allowed for assignment to an unknown environment in the case that the taxon is not shared by between the sink sample and the sources. In addition to our new data, we analyzed 16S sequence data generated from the 119 clones of 16S RNA gene PCR amplicons generated from an intestinal coprolite sample of the Tyrolean Iceman (Ötzi the Iceman) and the 49 clones generated from an intestinal coprolite sampled from an Austrian soldier killed in 1918 on a glacier [19].

The supplementary source tracking analyses using Yatsunenko et al's data [22] as a potential source include data for the primate gut microbiome [33] and the set of 88 soils [35]. These data were downloaded from MG-RAST [36] projects 850, 625 and 840–841, respectively. Phylogenetic assignments were made using Greengenes [18] at 97% identity.

## Supporting Information

Table S1 **QIIME 1.3.0 taxonomic assignments in detail.**
(XLSX)

Table S2 **The number of reads included in the analysis.** Trimmed data required a perfect match for forward and reverse primers and barcodes and a quality score of 25 or greater.
(DOCX)

Figure S1 **Flow chart of methods.** Steps framed by the green rectangle were performed in a laboratory dedicated to ancient degraded samples.
(TIF)

Figure S2 **Rarefaction curve and simulation values for the coprolite samples.** Due to the low diversity (number of OTUs) yielded by the Hinds Cave samples, they do not appear in the rarefaction curve.
(EPS)

Figure S3 **Bacterial communities clustered using Principal Coordinates Analysis of the unweighted UniFrac distance matrix.** Clusters were replicated using jackknifing to assess the degree of variation within the sample.
(EPS)

Figure S4 **Heatmap showing the log relative abundance of the most common (∼100) species.** The similarity between Rio Zape and rural African and primate sources is apparent. The source environment labels are on the right. “Primate-O” is from [22], “Primate-M” is from [32].
(EPS)

Figure S5 **Variation in source proportion predictions for coprolites and negative controls.** Each column in a plot represents the source mixture prediction from one of 25 random restarts of the SourceTracker Gibbs sampling procedure. The columns in each plot were reordered to place similar mixtures near one another in order to aid in visual interpretation. For example, sample CA10 was predicted to be completely “Unknown” in all but two of the random restarts; the remaining two were predicted to be mostly “Compost”. This indicates an identifiability issue with this sample: the model is unsure whether the sample is “Unknown” or “Compost”, and we have low confidence in the predictions for the sample. In contrast, we have high confidence in the predictions for the other samples as there is little variation in their source proportion estimates from the model.
(EPS)

Figure S6 **Variation in source proportion predictions for Tyrolean Iceman and 1918 soldier glacier mummy (sequence data published in** [**19**] **).** Each column in a plot represents the source mixture prediction from one of 25 random restarts of the SourceTracker Gibbs sampling procedure. The columns in each plot were reordered to place similar mixtures near one another in order to aid in visual interpretation. Predictions from the Tyrolean Iceman sample are consistent and we therefore have high confidence in them. Predictions from the 1918 soldier glacier mummy sample are low confidence, with the model sometimes predicting a moderate proportion of the primate gut source, sometimes a small proportion of the rural African child gut source, and sometimes a completely “Unknown” source.
(EPS)

Figure S7 **Evolutionary relationships of** ***Bifidobacterium*** **from Rio Zape 23.** Results are compared to eight previously published *Bifidobacterium* 16S rDNA sequences inferred by a Neighbor Joining tree.
(EPS)

Figure S8 **Frequency of Prevotellaceae.** Comparisons include published data from children from rural Africa and Europe [21] and the Rio Zape.
(EPS)

Figure S9 **Bayesian source-tracking results for Rio Zape.** For known sources, both Rio Zape samples assign primarily to the rural populations, Venezuela for ZA04 and Malawi for ZA23, with sample ZA04 also containing a predicted partial match to the modern non-human primate gut source, and a small partial match to US adults.
(EPS)

Fugure S10 **Bayesian source-tracking results for Rio Zape after considering age groups.** Both Rio Zape samples assign primarily to the rural populations, Venezuela Teen for ZA04 and Malawi adult for ZA23. Sample ZA04 also contains a predicted partial match to the modern non-human primate gut source, US adult and Venezuela adult. Sample ZA23 also contains a predicted partial match to modern non-human primate gut source, US adult and US infant.
(EPS)

Figure S11 **Rio Zape with varied rarefaction.** Trivial changes in proportions are observed when changing rarefaction depths.
(EPS)
