# Supplementary material for: Insights from Characterizing Extinct Human Gut Microbiomes
Source: PLoS One. 2012 Dec 12;7(12):e51146. doi: 10.1371/journal.pone.0051146 (PMC3521025; doi:10.1371/journal.pone.0051146)
Supplement: Table S2 — The number of reads included in the analysis. Trimmed data required a perfect match for forward and reverse primers and barcodes and a quality score of 25 or greater. (DOCX) [file pone.0051146.s002.docx]

**Table 2,** the number of reads included in the analysis. Trimmed data required a perfect match for forward and reverse primers and barcodes and a quality score of 25 or greater.

|  | **Location** | **Date (YBP)** | **Raw data** | **Trimmed data** | **Assigned data** | **Unique OTUs** |
| --- | --- | --- | --- | --- | --- | --- |
| BE04 | Hinds Cave - USA | 8000 | 27478 | 45 | 45 | 25 |
| BE21 | Hinds Cave - USA | 8000 | 31113 | 47 | 34 | 10 |
| CA10 | Caserones – Chile | 1600 | 14640 | 3032 | 3028 | 50 |
| CA18 | Caserones – Chile | 1600 | 30695 | 6786 | 6784 | 42 |
| ZA04 | Rio Zape – Mexico | 1400 | 31222 | 6162 | 5668 | 197 |
| ZA23 | Rio Zape – Mexico | 1400 | 33252 | 6854 | 6735 | 249 |
| Hum | * | Modern | **** | 2365 | 2226 | 118 |
| Pri | ** | Modern | **** | 1493 | 1181 | 173 |
| EX | *** | Modern | 47818 | 8218 | 7881 | 201 |

* Pooled dataset from three modern humans (two from Gill 2006 and 399 sequences from one human sample retrieved from NCBI accession numbers GU939195.1 to GU939593.1)

** Pooled dataset from one Bonobo (BNO), one Chimpanzee (Chimp), two Gorillas (GOR and GORSD), one Marmoset (ML) and one Orangutan (ORANG)

*** Negative contamination controls used during DNA extraction

**** Number of raw data reads was not provided for these previously published data
