# Supplementary figures and images for: Insights from Characterizing Extinct Human Gut Microbiomes
Source: PLoS One. 2012 Dec 12;7(12):e51146. doi: 10.1371/journal.pone.0051146 (PMC3521025; doi:10.1371/journal.pone.0051146)

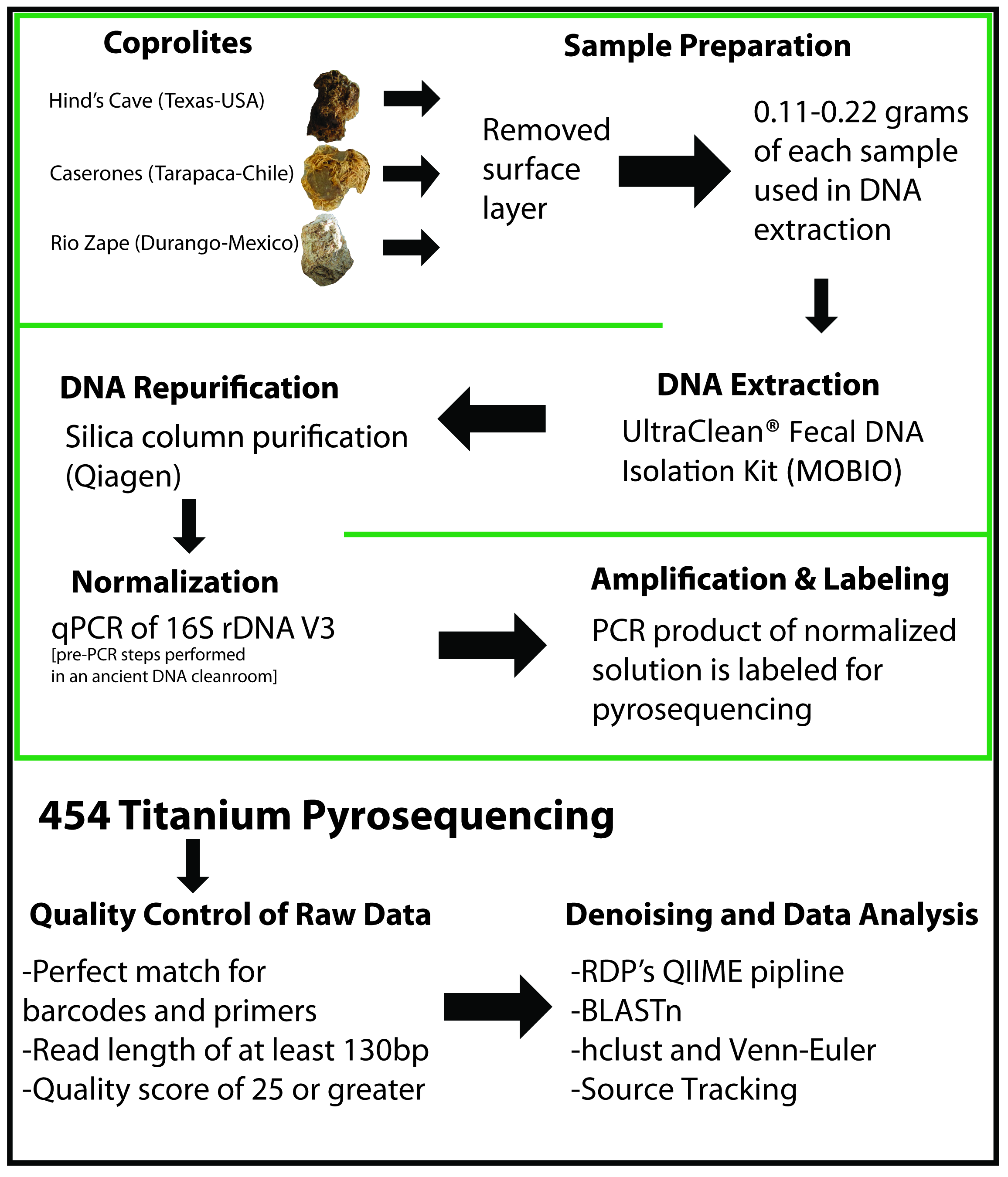

Supplement: Figure S1 — Flow chart of methods. Steps framed by the green rectangle were performed in a laboratory dedicated to ancient degraded samples. (TIF) [file pone.0051146.s003.tif]
